# Association of reduced right ventricular global and regional wall motion with abnormal right heart hemodynamics

**DOI:** 10.1186/1532-429X-17-S1-P317

**Published:** 2015-02-03

**Authors:** Lakshmi Muthukumar, Lynette J Duncanson, William Schapiro, Jeannette McLaughlin, Alistair Young, Jie J Cao

**Affiliations:** 1 Cardiac Imaging, St. Francis Hospital, Port Washington, NY, USA

## Background

The relationship of right ventricular (RV) regional and global systolic function to right heart hemodynamics is not well understood. In this study we used MRI feature tracking to assess regional RV wall motion, cine MR to evaluate global RV function and examined the relationship of regional wall motion and global RV function to right heart hemodynamics.

## Methods

Fifty patients undergoing clinically indicated right heart catheterization were prospectively recruited to a research CMR within 5 hours of catheterization. Majority of the heart failure cases were due to left heart failure. SSFP cine images were acquired to evaluate RV regional and global function. RV longitudinal and long axis radial strains were derived from the 4-chamber cine, and mid wall septal circumferential and radial strains from the short-axis plane in mid ventricle using CIM *feature* tracking software (Auckland, NZ). Right heart hemodynamics was assessed during catheterization.

## Results

Mean age was 64±13 years, mean RV ejection fraction (EF) 51±13%. Reduced longitudinal and radial displacement in 4 chamber view was significantly associated with reduced RVEF, r=-0.621 (p<0.001) and r=0.346 (p=0.014), respectively. Similarly, in short axis plane reduced septal circumferential and radial strain were also significantly correlated with RVEF, r=-0.488 (p=0.001) and r=0.527 (p<0.001) respectively. In regression analysis reduced RVEF had strongest association with increased pulmonary wedge pressure (r=-0.622, p<0.001) in univariate analysis and in multivariate analysis (p<0.001) after adjusting for all the right heart hemodynamic parameters. Pulmonary wedge pressure contributed to 41% of RVEF variation in this cohort with predominant left heart failure. While also significantly correlated with pulmonary wedge pressure, regional wall motion including longitudinal strain, septal circumferential and radial strain had strongest association with mean pulmonary arterial pressure, r=0.577 (p<0.001), r=0.440 (p=0.002) and r=-0.451 (p=0.001), respectively.

## Conclusions

It is feasible to use MR feature tracking to characterize RV regional wall motion. Reduced global and regional RV systolic functions are associated with right heart hemodynamic abnormalities. In patients with predominant left heart failure, pulmonary wedge pressure contributes importantly to RVEF variation.

## Funding

None.

